# A multi-technique and multiscale comparative study on the efficiency of conservation methods for the stabilisation of waterlogged archaeological pine

**DOI:** 10.1038/s41598-024-58692-6

**Published:** 2024-04-15

**Authors:** Ingrid Stelzner, Jörg Stelzner, Björn Fischer, Elias Hamann, Marcus Zuber, Philipp Schuetz

**Affiliations:** 1 Leibniz-Zentrum für Archäologie, Mainz, Germany; 2 FISCHER GmbH, Raman Spectroscopic Services, Meerbusch, Germany; 3 Institute for Photon Science and Synchrotron Radiation, Karlsruhe Institute of Technology, Eggenstein-Leopoldshafen, Germany; 4 Lucerne University of Engineering and Architecture, Horw, Switzerland

**Keywords:** Structured-light 3D scanning, Synchrotron micro-computed tomography, Raman imaging, Waterlogged archaeological wood, Conservation, Biotechnology, Plant sciences, Materials science

## Abstract

Archaeological wood can be preserved in waterlogged conditions. Due to their degradation in the ground, these archaeological remains are endangered after their discovery, since they decay irretrievably during drying. Conservation measures are used to preserve waterlogged archaeological objects, maintaining their shape and character as much as possible. However, different methods have been developed leading to varying results. This study compares their effectiveness in order to clarify their mode of action. The methods including alcohol-ether resin, lactitol/trehalose, melamine formaldehyde, polyethylene glycol impregnation prior to freeze–drying, saccharose and silicone oil were assessed by analysing mass changes and volume stability using structured-light 3D scanning. The state of the conserved wood samples including the spatial distribution of the conservation agent was examined using synchrotron micro-computed tomography. Raman spectroscopy was used to observe the agent´s spatial distribution within the cells. The findings demonstrated that melamine formaldehyde stabilises the degraded cell walls. The lumens are void, as in the case with alcohol-ether resin, while polyethylene glycol, silicone oil, saccharose and lactitol/trehalose also occupy the lumens. It is assumed that the drying method has an effect on the distribution of the solidifying agent. The knowledge gained affords insights into the mechanism of conservation methods, which in turn accounts for the varied outcomes. It also allows conclusions to be drawn about the condition and stability of conserved museum objects and serves as a starting point for the further development of conservation methods.

## Introduction

Organic material such as wood, can be preserved in temperate climate zones in water-saturated soils. Wooden artefacts, for example, can be preserved for centuries or even millennia, providing valuable clues to past cultural practices and way of life. The study of these artefacts enables the reconstruction of past cultures, offering an intriguing glimpse into the past. These wooden artefacts are kept in museums and collections^1^. Waterlogged archaeological wood, once recovered, is highly fragile and can be completely destroyed in a matter of hours. This fragility is due to the gradual degradation of archaeological wood during ground deposition, starting from the outer regions^2–6^. Such degradation has greatly altered the structure and composition of the objects. The degradation of the secondary cell wall’s cellulosic components by microorganisms, such as fungi and bacteria, is noteworthy. Moreover, lignin can be broken down through oxidation and hydrolysis. In this manner, one tracheid is degraded while its neighbouring cell remains undamaged. This scenario is widespread, leading to the ultimate stage of deterioration where only the middle lamella´s skeletal frame upholds the structure together. The secondary cell wall, which is primarily responsible for maintaining stability in undamaged wood, contains degraded lignin and bacterial slime throughout^7^.

When wood dries, capillary forces occur due to the high surface tension of the water as it evaporates. The resulting effect is the shrinkage of the cell walls and collapse of the cells. Depending on the degree of degradation, only a fraction of the wood volume is preserved^8–10^.

The preservation of the unique wooden sources for archaeological research have been of concern since the nineteenth century^11^, leading to the development of methods to avoid damaging drying^12^. Precautions and conservation measures aim to preserve the cultural heritage while respecting its significance, including accessibility for present and future generations^13^. Therefore, conservation agents must effectively permeate, adsorb and stabilize the cell wall and or the lumen, ensuring that shrinkage is prevented.

The mode of action of agents for wood conservation is based on two distinct principles^14,15^. Firstly, impregnation involves filling the degraded wood (lumina, cell wall and microcapillaries) with a conservation agent, which then solidifies (e.g. through cooling, chemical precipitation, polymerisation or condensation polymerisation) to provide structural reinforcement and mechanical stability. This process protects the wood from reactions during drying. The second principle is when solely the micropores and pores of the cell wall are filled and strengthened using a bulking agent. This makes the cell wall more robust and less prone to drying stress. Moreover, shrinkage is minimised, and adsorption and desorption with air are reduced. Consequently, bulking agents are required to have a diameter < 10 nm in diameter. Nonetheless, if the secondary cell wall is significantly degraded, there may be nothing to fill but only to stabilise.

It is crucial to avoid wood collapse while drying. One way to reduce capillary tension is by using solvents with surface tensions lower than that of water (e.g. acetone, ethanol, ether)^12^. Freeze–drying (sublimating water as gas directly from the solid state) is also employed^16^. Eliminating the liquid phase will eliminate the forces of capillary tension. Conservation agents are added to prevent shrinkage and act as a cryoprotectant to prevent volume expansion during freezing^16^.

Conservation methods with significant relevance comprise alcohol-ether resin, Kauramin 800® (melamine formaldehyde), lactitol/trehalose, freeze-drying of polyethylene glycol (PEG)-impregnated wood, saccharose and organosilicon compounds^12,17,18^. The Anti-Shrink Efficiency (ASE) measurements demonstrate a substantial variation in the ability of these methods to stabilise finds^18–20^. This study analyses the factors contributing to the varied efficiencies of conservation agents and addresses three critical questions: (1) Is the quantity of conservation agents integrated into the structure a paramount factor for achieving volume stabilization? (2) Does the mode of action (bulking/impregnation) significantly influence volume stability? (3) Is the consolidant compatible with the cell wall?

Wood conservation poses challenges due to the wood´s inherent heterogeneity of the wood in morphology and chemical composition. This heterogeneity is influenced by factors such as the wood species, burial environment and type of decay. To maintain consistency in our findings, we refined our research to a single object. To address the initial query (1), we recorded simultaneous mass and dimensional changes by performing weight measurements and implementing a structured-light 3D scanner before and after conservation. To investigate the link between the results and the mode of action of the conservation agent (2), high-resolution structural examinations were carried out using synchrotron micro-computed tomography (synchrotron µCT). For the third query (3), confocal Raman imaging was utilised to trace the conservation agent within the cellular wall.

## Material and methods

### Samples

Samples from the reference collection of the Leibniz-Zentrum für Archäologie, LEIZA (formerly the Römisch-Germanisches Zentralmuseum, RGZM) were utilised for this investigation^18^. The selected samples were obtained from an undated U-shaped water pipe located in Stralsund, Germany (number V03). The pipe was dissected into six longitudinal strips and resized to approximately of 12 cm × 7 cm × 9–12 cm (length × width × height). The type of wood used was identified as *Pinus* sp. The water pipe exhibits mixed degrees of degradation. The degradation is observed from the exterior to the interior, elucidating the strong decay of the outer area and the well-preserved inner region. The gradient shift in morphology towards the core is similarly highlighted by the Maximum Water Content (MWC) and the Basic Density (BD)^20–24^. These specific properties were ascertained through the measurement of the underwater weight (w_sub_) and the wet weight (w_wet_) and calculated (1)^25^. W_sub_ denotes the recorded weight of an object in water, which is less than its actual weight in air due to the force of buoyancy. Wet weight (w_wet_) pertains to the weight of a sample that has been saturated with water measured in the air. The bulk density (BD) was calculated as 251 kg/m^3^ (2)^26^, after obtaining a MWC of 332%.

1 $$MWC = \frac{{W_{wet} - \left( {3 \times W_{sub} } \right){ }}}{{3 \times W_{sub} }} \times 100 \left[ \% \right]$$

2 $$BD = \frac{100}{{66.7 + MWC}} \left[ {\frac{{\text{g}}}{{{\text{cm}}^{3} }}} \right]$$

To measure the changes in weight and dimensions before and after conservation, five samples were conserved using each of the nine methods specified in Table 1 at various collaborators. These measures include the alcohol-ether-resin method^27,28^, Kauramin 800® (melamine formaldehyde)^29^, lactitol/trehalose^30–32^, freeze-drying after impregnation with an aqueous solution of PEG at different molecular weights. PEG 2000 (PEG1)^33^, PEG 400 and 4000 (PEG2)^25^, PEG 400, 1500 and 4000 (PEG3)^18^, saccharose^34,35^ and silicone oil^36,37^ were used in the experiment. Furthermore, one of the reference samples was only air-dried.

**Table 1:** Detailed protocol of the conservation treatments.

Alcohol ether resin (Swiss National Museum, Zürich, Switzerland) (AlEt)
First, the water in the waterlogged samples was exchanged with ethanol and then the ethanol with diethyl ether. This was performed in multiple steps. Then the samples were soaked in a solution of 70.7% diethyl ether, 16.1% dammar resin, 6.4% rosin, 3.2% dienol D102, 3.2% rhizinus oil, 0.4% PEG 400. Drying was performed by evaporation of the diethyl ether in a vacuum vessel. The surface was then consolidated with 3% Paraloid B72 solution in acetone
Melamine formaldehyde (Leibniz-Zentrum für ArchäologieMainz, Germany) (K800)
After soaking the specimens in distilled water, the specimens were impregnated at room temperature in a solution of 25% Kauramin 800®, BASF (72 L resin + 210 L deionised water, 3.6 L urea, 7.2 L triethylene glycol). The samples were taken from the solution before polycondensation occurs. Curing of the impregnated wood was performed in a heating cabinet at 60 °C. Afterwards the samples were slowly air-dried. The surface was treated with linseed oil varnish
Lactitol/trehalose (Brandenburgisches Landesamt für Denkmalpflege, Zossen, Germany) (LaTr)
Impregnation started with a 30% aqueous solution of lactitol/trehalose (9:1), which was concentrated monthly in 10% steps until 70%. When necessary, a biocide (0.1% Bioban 404) was added to the solution. The bath temperature was 55 °C. After the removing the samples from the bath, they were dusted with crystalline lactitol monohydrate and dried in a heating oven over a period of one week. After drying, the surface was cleaned by dabbing with damp cloths
Freeze–drying after PEG 2000 impregnation (Nationalmuseet, Copenhagen, Denmark) (PEG1)
Conservation by a solution of PEG followed by freeze–drying (PEG1). The treatment started with a 10% PEG 2000 solution. The concentration was concentrated to 40% in 10% steps at room temperature. Freeze–drying was performed in a cooled chamber (pprox.. − 30 °C). Excess of PEG on the surface was removed with a soft brush and ethanol. Further surface stabilization was achieved with a solution of 25% PEG 2000 in ethanol
Freeze–drying after PEG 400 and 4000 impregnation (Brandenburgisches Landesamt für Denkmalpflege, Zossen, Germany) (PEG2)
The samples were washed in demineralized water. The PEG-solution was prepared in demineralized water (PEG 400 and PEG 4000) and the final concentration was adapted according to the condition of the wood at 10% PEG 400 and 25% PEG 4000 (PEGcon)^25^. Subsequently, the concentration was increased in 5% steps until the final concentration was reached and kept constant. In a second step, PEG 4000 was added. The concentration was increased in 5% steps until the final concentration was reached. Then the samples were precooled at 5 °C and frozen at − 25 °C to − 35 °C and freeze-dried at approx: − 30 °C in cooled chamber of a freeze–drying plant
Freeze–drying after PEG 400, 1500 and 4000 impregnation (Archäologische Staatssammlung, Munich, Germany) (PEG3)
The samples were first soaked in demineralized water. Then, at room temperature, an 11% solution was started and concentrated to 15% PEG 400. Then the solution was heated to 40 °C and concentrated from 16% to 20.5% with PEG 1500. From 20.5% to 27.5% the solution was concentrated with PEG 4000 at 40 °C. After impregnation, samples were washed and wrapped in cellulose and frozen (− 25 to − 35 °C) until freeze–drying in a cooled chamber (approx: − 30 °C). Excess PEG was removed with a brush and ethanol
Saccharose (Sächsisches Landesamt für Archäologie, Dresden, Germany) (Sac)
The solution was concentrated in 10% steps from 10 to 60% at room temperature. Then slow, controlled air-drying was carried out in microperforated bags. The crystalline sugar on the surface was removed with damp sponge. If necessary biocide addition composed of 0.6%, sodium benzoate (E211), 0.5% Parmetol K40 (mixture of Isothiazols, Schülke & Mayr GmbH, Norderstedt, Germany), 0.5% Quartasept Plus (a combination of quaternary ammonium compounds and amine derivatives, Schülke & Mayr GmbH, Norderstedt, Germany) and 0.02% Tallofin OT (bio-dispersant, Solenis LLC Wilmington, DE, USA)
Silicone oil (University of Texas, Texas, USA) (Sil)
The samples were conserved with silicone oil (siloxane oil, CAS number 70131–67-8)^37^. The water was first replaced by ethanol and then by acetone. With a solution of 80% silicone oil (SFD1 (66%) + SFD5 (34%)—silanol functional polydimethylsiloxanes “PDMS”) and 20% crosslinker MTMS (methyltrimethoxysilane), the samples were impregnated under normal atmospheric conditions. The polymerization of the impregnation solution was triggered by a gaseous catalyst: DBTDA (dibutyl diacetate)

For purposes of structural analyses, one sample of each conservation method was chosen. In order to investigate how the conservation agent was incorporated, a core sample with a diameter of 5 mm was obtained from the highly degraded edge region using an increment borer. Two samples, each with a length of approximately 5 mm, were extracted from the total drill sample for synchrotron µCT analysis and Raman imaging. For synchrotron µCT analysis, the sample diameter was decreased to 2 mm.

Before conducting Raman analyses, the sample preparation was thoroughly evaluated. The primary objective was to avoid altering the conservation agent, while obtaining a smooth surface for precise measurements. Grinding the surface was initially tried, but it was evident that this method did not yield a surface that was smooth enough, and there was a potential risk of altering the conservation agent. As an alternative the preparation of thin sections was assessed. However, this was not possible for some samples due to their delicacy or hardness. As a result, it was decided to embed the samples before sectioning on a microtome to ensure a suitable surface for Raman analysis. The process involved embedding around 5 mm wide segments of the drill core in Technovit 7100 (Kulzer, Germany). The samples were initially positioned in a negative mould and then resin was allowed to infiltrate them. In the following step, the negative mould was filled. After curing, the samples were mounted on a support (Histobloc, Kulzer Germany) using Technovit 3040 (Kulzer, Germany). Thin sections of roughly 5 µm thickness were cut from these blocks employing a microtome acquired from the Swiss Federal Institute for Forest, Snow and Landscape Research (WSL) located in Birmensdorf, Switzerland. This method gave the best results ensuring that the samples presented a smooth surface suitable for precise Raman analyses.

### Calculation from mass and volume changes

The difference between the weight of the conserved sample (w_cons_) and the oven-dried wood (w_wood_) indicates the amount of the conservation agent absorbed. The weight of the conserved sample is readily measurable by weighing. W_wood_ is estimated non-destructively using the ‘Archimedes’ principle (3)^25^ whereby the weight of the wet sample prior to conservation is used. Assuming the sample to be fully saturated with water, the wood sample displaces water according to its volume and a density of 1 $\frac{{\text{g}}}{{{\text{cm}}}^{3}}$. The wood is lighter due to the displacement of the water in relation to its volume. The actual weight of the sample can be calculated based in the known density of wood of 1.5 $\frac{{\text{g}}}{{{\text{cm}}}^{3}}$. The Percentage Weight Gain (WPG) (4)^38–40^ resulting from conservation can be calculated by assuming an equilibrium moisture content of 6% in the conserved wood at the atmosphere (equivalent to a relative humidity of approximately 30%), which has been subtracted from w_cons_.^41^

3 $$w_{{{\text{wood}}}} = { }3{ } \times w_{sub} { }\left[ {\frac{{\text{g}}}{{{\text{cm}}^{3} }}} \right]$$

4 $$WPG = { }\frac{{w_{cons} - w_{{{\text{wood}}}} }}{{w_{{{\text{wood}}}} }} \times 100{ }\left[ {\text{\% }} \right]$$

The ASE, which is a metric used to determine the effectiveness of a wood conservation method in preventing shrinkage^15,42^ was determined by measuring the volume of wet samples before treatment and dry samples after treatment using a structured-light 3D scanner^20,43^. Therefore, the wet samples were captured before conservation and in dry condition after conservation with an ATOS III Rev. 01 scanner from GOM (a ZEISS company), with a field of view of 500 mm × 500 mm × 500 mm and a point distance of 0.25 mm. The 3D mesh with a closed surface was obtained by processing the scans in ATOS Version v6.2 software through Python scripts with similar parameters. To determine the sample’s shrinkage, by employing to control the workflow and obtain a reduced 3D mesh with a closed surface. On the basis of the volume data before (V_wet_) and after conservation (V_dry_), the shrinkage of the sample was calculated (5). Subsequently, the ASE was determined by calculating the shrinkage of a non-conserved control sample (S_0_) and the shrinkage of the conserved sample (S_con_) (6).

5 $$S = \frac{{V_{wet} - V_{dry} }}{{V_{wet} }} \times 100 \left[ \% \right]$$

6 $$ASE = \frac{{S_{0} - S_{con} }}{{S_{0} }} \times 100 \left[ \% \right]$$

### Synchrotron µCT

Propagation-based phase contrast synchrotron µCT scans were performed at the imaging cluster of the Karlsruhe Institute for Technology (KIT) light source using a parallel polychromatic X-ray beam produced by a 1.5 T bending magnet which was spectrally filtered by 0.7 mm aluminum. A rapid and indirect detector system was used which comprised a 12 µm thick LSO:Tb scintillator^44^ and a diffraction limited optical microscope (Optique Peter, Lentilly, France) coupled to a 12 bit pco.dimax S4 high speed camera with 2016 × 2016 pixels using an optical magnification of 10x, resulting in an effective pixel size of 1.22 µm and a field of view of approximately 2.4 mm × 2.4 mm. Scans were performed by taking 3000 projections over 180° sample rotation at 70 frames per second at a propagation distance of ~ 7 cm. The control system concert^45^ was used for automated data acquisition and online reconstruction of tomographic slices for data quality assurance. The UFO/tofu frameworks^46,47^ were employed for both online and final data processing including phase retrieval based on the transport of intensity equation (TIE)^48^ and tomographic reconstruction. Analyses of the data were conducted using VGStudio MAX 3.4© software (Volume Graphics, Heidelberg, Germany).

### Confocal Raman Microscopy

Confocal Raman Microscopy (CRM) was performed using an alpha 300 R Raman microscope (WITec, Ulm, Germany). The system was equipped with a WITec UHTS 300 spectrometer and an Andor iDus Deep Depletion charge-coupled device (CCD) camera, which was cooled to a temperature of − 60 °C. For excitation, a single-mode laser with a wavelength of 532 nm was utilized. To achieve an average spectral resolution of 3.8 cm^−1^/pixel, a reflection grating with 600 lines/mm was employed. Raman images were acquired using a Zeiss EC Epiplan-Neofluar HD 20 × /0.5 or Zeiss EC Epiplan-Neofluar Dic 50 × /0.8 microscope objective, according to sample roughness. The laser power was adjusted between 2 and 5 mW depending on the susceptibility of the samples to laser radiation. The measured areas ranged from 50 × 50 µm to 90 × 90 µm, with a spatial resolution of 0.5–1.0 µm. The Raman images were acquired with an integration time of 0.2–1.0 s per spectrum. Furthermore, light microscopy bright-field images of the samples were captured using a Zeiss EC Epiplan-Neofluar Dic 100 × /0.9 microscope objective. The images covered an area of 108 × 68 µm^2^.

The Raman images were compiled using the True Component Analysis module of the WITec FIVE software (version 5.3.18.110, WITec, Ulm, Germany). After cosmic ray removal, baseline subtractions of all spectra were performed to remove fluorescence. This was done by subtracting the background using rounded shapes that approach the spectrum pixel by pixel from below. To compile the Raman images, the entire measured hyper spectral data set is described by a linear combination of already known spectra (base spectra), which are obtained by an averaging procedure from the same hyper spectral data set. Each component points in a different direction of the high-dimensional vector space. These spectra or a linear combination of them form a basis that only describes a subspace of the entire vector space. With this basis, all measured spectra can be described as linear combinations. The Raman spectra were plotted using the software Origin Pro 2021. A Savitzky-golay filter (2nd order, 7 points) was applied to smooth the spectra and subsequently normalized.

## Results

### Changes in weight

**Figure 1:**
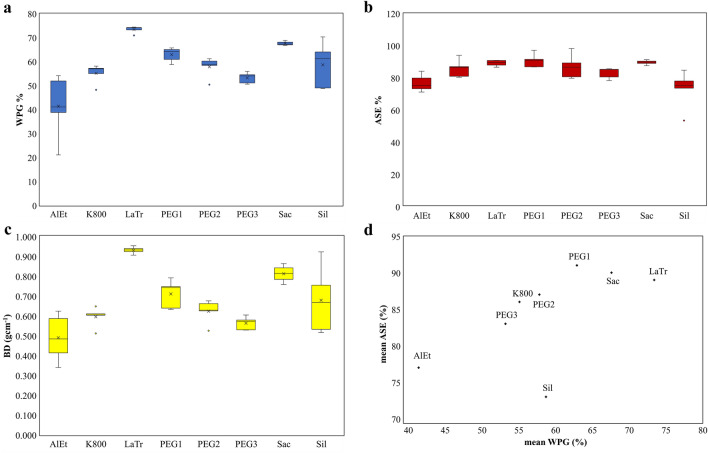
Each data item reflects five samples (n = 5) for each conservation treatment, including alcohol-ether resin (AlEt), Kauramin 800® (K800), lactitol/trehalose (LaTr), PEG 2000 (PEG1), PEG 400 and PEG 4000 (PEG2), as well as PEG 400, PEG 1500, and PEG 4000 (PEG3) prior to freeze–drying, saccharose (Sac), and silicone oil (Sil). (**a**) Weight per cent gain (WPG, %), (**b**) Anti-Shrink Efficiency (ASE, %), and (**c**) bulk density (BD, g/cm^3^) are drawn against their treatment. (**d**) Correlation between mean value of 5 samples WPG (%) and ASE (%).

The lactitol/trehalose treatment achieved the highest WPG (73.5 ± 1.4%, cf. Fig. 1a) when impregnated with a 70% solution, with saccharose (67.7 ± 0.9%) using a 60% solution and PEG1 (63.0 ± 2.9%) treated with a 40% solution following closely behind. Additionally, silicone oil displayed a noteworthy WPG of 58.8 ± 9.5%. Impregnation with PEG2 at a 35% solution illustrated a WPG of 57.9 ± 4.3%. Kauramin 800® yielded a value of 55.2 ± 4.0% using a 25% solution, whereas PEG3 reached a WPG of 53.3 ± 2.3% with a 28% solution. The alcohol-ether resin method achieved a WPG of 41.5 ± 13.1% with a 29% solution. However, the WPG that expresses the consolidant uptake is also prone to variations. Notably, the samples conserved with alcohol-ether resin or silicone oil exhibit high standard deviations (cf. Table 2). Certain variations, particularly in the samples treated with alcohol-ether resin and Kauramin 800®, may be ascribed to the use of a surface coating (cf. Table 1).

**Table 2:** The concentration of the impregnation solution (c), the weight percent gain (WPG), the dry bulk density (BD), and the shrinkage (S) is displayed for the conserved pine samples.

Method		Pine (n = 5)				Several species^20^ (n = 7–10)
	c (%)	WPG (%)	BD (g cm^-1^)	S (%)	ASE (%)	ASE (%)
AlEt	29	41.5 ± 13.1	0.491 ± 0.118	5.3 ± 1.2	77.0 ± 5.2	90.7 ± 6.6
K800	25	55.2 ± 4.0	0.597 ± 0.050	3.2 ± 1.3	86.0 ± 5.5	95.5 ± 5.0
LaTr	70	73.5 ± 1.4	0.933 ± 0.019	2.5 ± 0.5	89.4 ± 2.1	81.2 ± 10.7
PEG1	40	63.0 ± 2.9	0.712 ± 0.071	2.1 ± 1.0	90.9 ± 4.3	96.3 ± 7.0
PEG2	35	57.9 ± 4.3	0.625 ± 0.059	3.0 ± 1.7	87.1 ± 7.5	93.9 ± 8.4
PEG3	28	53.3 ± 2.3	0.565 ± 0.033	3.9 ± 0.8	83.2 ± 3.4	93.1 ± 6.6
Sac	60	67.7 ± 0.9	0.814 ± 0.046	2.4 ± 0.4	89.9 ± 1.6	78.7 ± 10.0
Sil	100	58.8 ± 9.5	0.680 ± 0.168	6.2 ± 2.7	73.2 ± 11.7	81.1 ± 7.4

The Anti-Shrink Efficiency (ASE) is shown for both, the samples of this study and reference samples from literature^20^. These samples underwent various treatments, including alcohol-ether resin (AlEt), Kauramin 800® (K800), lactitol/trehalose (LaTr), PEG 2000 (PEG1), PEG 400 and PEG 4000 (PEG2), as well as PEG 400, PEG 1500, and PEG 4000 (PEG3) prior to freeze–drying, saccharose (Sac), and silicone oil (Sil).

### Changes in volume

The samples treated with alcohol-ether resin displayed an average ASE of 77.0 ± 5.2% (cf. Fig. 1b), whereas the Kauramin 800® treated samples achieved an ASE of 86.0 ± 5.5%, and the lactitol/trehalose treated samples reached a mean value of 89.4 ± 2.1%. Similarly, the samples treated with PEG1 showed an average ASE of 90. 9 ± 4.3%, the PEG2 samples had an ASE of 87.1 ± 7.5% and the PEG3 samples displayed an ASE of 83.2 ± 3.4%. The samples treated with saccharose achieved an average ASE of 89.9 ± 1.6%, while the silicone oil conservation method yielded a mean value of 73.2 ± 11.7% for the ASE. It is important to note that the observed trends appear to differ from the shrinkage measurements obtained in previous experiments (cf. Table 2)^20^. The earlier measurements encompassed a broader range of samples (with n values ranging from 7 to 10) and included additional factors, such as the type of wood. The alcohol-ether resin technique demonstrated significantly better ASE values, attaining 90.7 ± 6.6%. Similarly, Kauramin 800® recorded an ASE of 95.5 ± 5.0%. Contrastingly, lactitol/trehalose accomplished a lower value of 81.2 ± 10.7%. PEG1 yielded superior results, achieving an average ASE of 96.3 ± 7% in comparison to PEG2 with 93.9 ± 8.4% and PEG3 with 93 ± 6.6%. However, saccharose only reached 78.7 ± 10.0% ASE. Additionally, silicone oil produced better results with 81.1 ± 7.4% ASE. These results indicate that the effectiveness of the treatment could be related to the wood genus. The pine samples showed considerable stabilising effects due to the sugar-based treatments, including saccharose and lactitol/trehalose, with a low standard deviation. Nevertheless, the values in the other series were lower, and the standard deviation was significantly higher, indicating lower reliability. All other conservation methods delivered significantly unfavourable outcomes in the pine test series.

### Dry bulk density

The BD was ascertained by computing the mass per unit volume of the samples, and noteworthy variances were observed among the diverse techniques employed (cf. Fig. 1c). The samples treated with alcohol-ether resin manifested a density of 0.491 ± 0.118 g/cm^3^, whereas those conserved with Kauramin 800® registered a density of 0.597 ± 0.05 g/cm^3^. Notably, the samples conserved with lactitol/trehalose recorded a BD of 0.933 ± 0.019 g/cm^3^. PEG1 resulted in a BD of 0.712 ± 0.071 g/cm^3^ and similarly, PEG2 led to a BD of 0.625 ± 0.059 g/cm^3^. However, PEG3 resulted in a lower BD of 0.565 ± 0.033 g/cm^3^. Samples impregnated with saccharose exhibited a comparatively higher BD of 0.814 ± 0.046 g/cm^3^. In comparison, silicone oil produced a lower BD of 0.680 ± 0.168 g/cm^3^. By contrast, the untreated and air-dried reference sample had the lowest BD of 0.268 g/cm^3^. The samples conserved using lactitol/trehalose exhibited the highest density, followed by those conserved using saccharose, silicone oil, PEG1, PEG2, PEG 3, Kauramin 800® and alcohol-ether resin. These findings concur with those derived from the WPG analysis. The samples treated with silicone oil exhibit the most significant data scatter, followed by those treated with alcohol-ether resin, PEG-based conservation methods, and saccharose. In contrast, lactitol/trehalose and Kauramin 800® treatments yield consistent and reliable results (cf. Table 2).

### Synchrotron µCT

**Figure 2:**
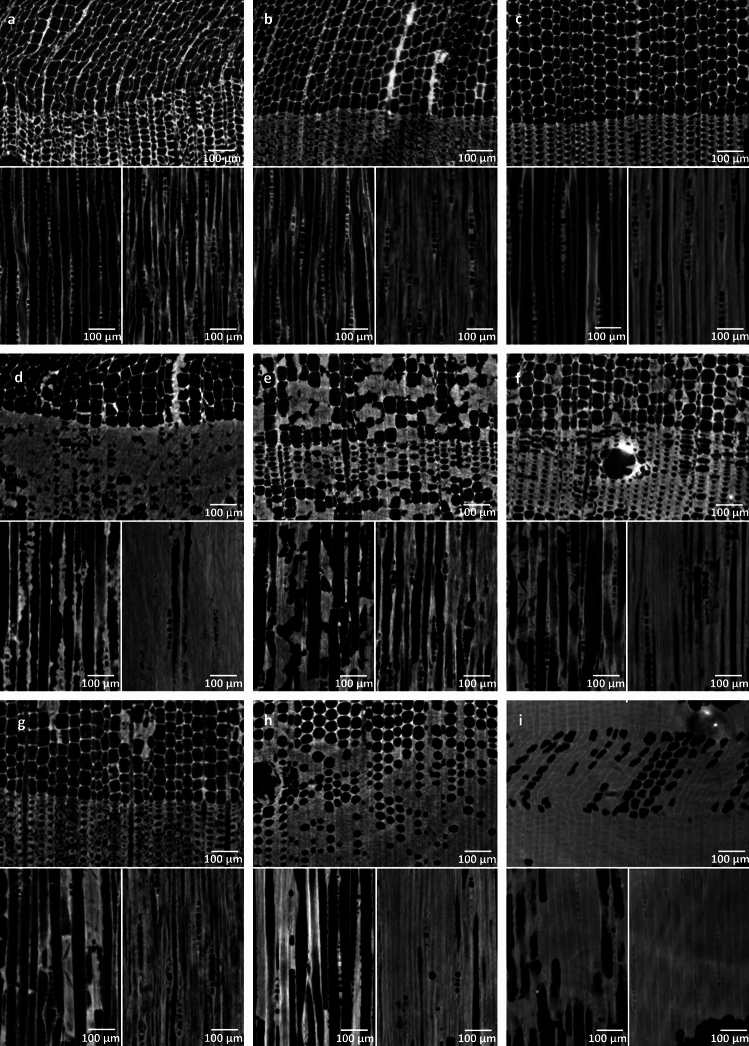
Synchrotron µCT imaging of the wood structure of (**a**) an untreated reference sample, a sample conserved using (**b**) alcohol-ether resin, (**c**) Kauramin® 800, (**d**) lactitol/trehalose, (**e**) PEG1, (**f**) PEG2, (**g**) PEG3, (**h**) saccharose, and (**i**) silicone oil. Note, that deformations in the sample conserved with silicone oil may be the result of preparation, since the sample was very soft.

The synchrotron µCT analyses confirmed the changes in mass and volume mentioned earlier. Notably, Fig. 2a demonstrates that the untreated and air-dried sample has a significantly distorted and crumpled form, with the cell walls contracting, compressing, and partially deforming. In contrast, all conservation methods resulted in cells with a more regular shape and thicker cell walls. It is worth pointing out that the secondary cell wall of the sample conserved with alcohol-ether resin showed particularly pronounced shrinkage, as shown in Fig. 2b. Similarly, Fig. 2g illustrates a comparable shrinkage pattern in the sample conserved with PEG3.

**Figure 3:**
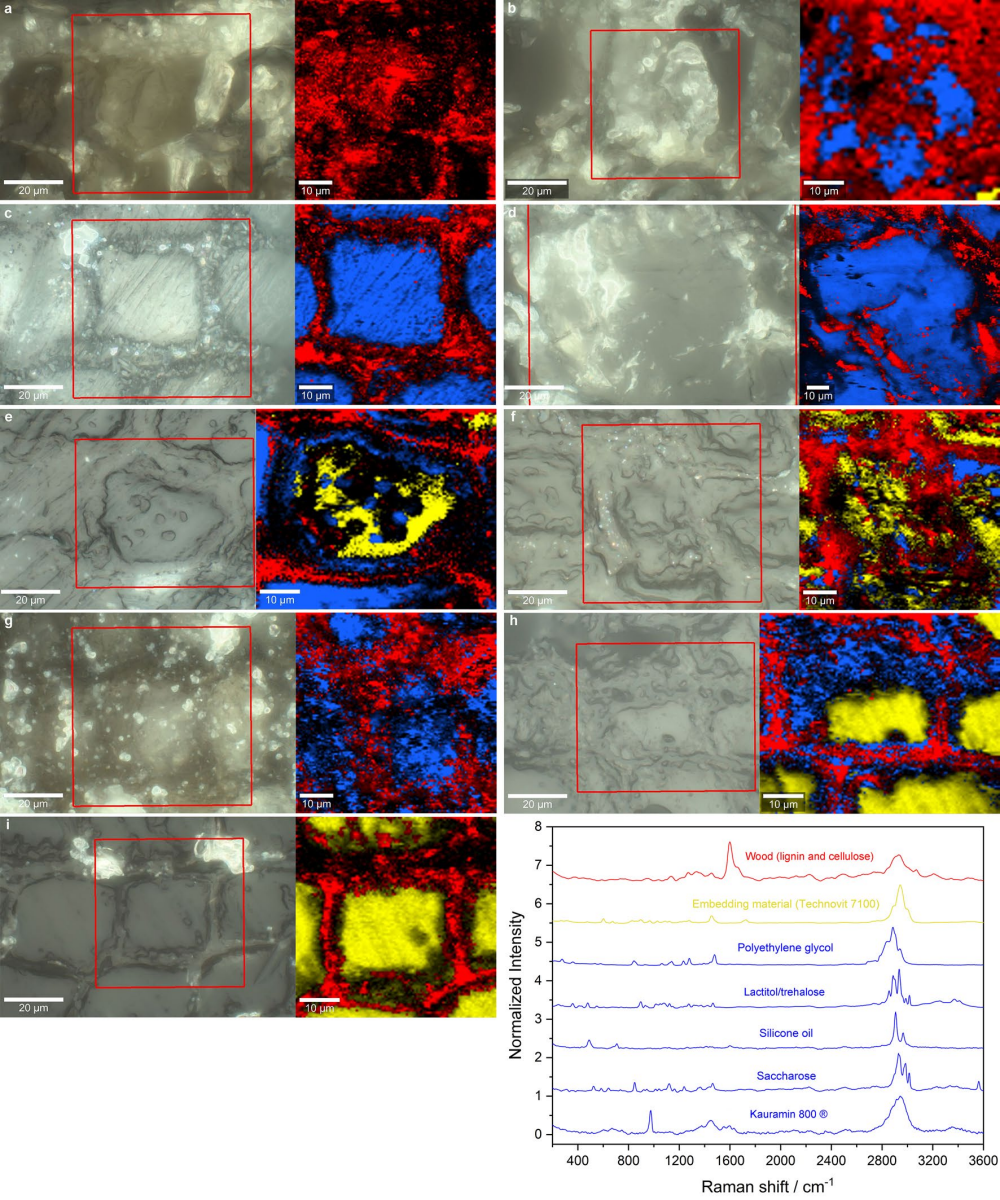
Raman imaging showing the distribution of the different conservation agents within the structure of archaeological pine wood samples using the methods (**a**) alcohol-ether resin, (**b**) Kauramin800®, (**c**) lactitol/trehalose, (**d**) PEG1, (**e**) PEG2, (**f**) PEG3, (**g**) saccharose and (**h**) silicone oil. In comparison, an untreated reference (**i**) is shown.

The comparison of synchrotron µCT images of the conserved samples reveals distinct patterns of consolidant deposition within the wood. Notably, both Kauramin 800® and the resin mixture employed in the alcohol-ether resin method seem to mainly penetrate or stabilize the cell walls, whilst the rest of the consolidants more or less fill the cell lumina. The distribution of consolidants and the degree of uniform structure stabilization significantly differ across the different conservation treatments. For example, the wooden structure of the silicone treated sample is entirely filled with silicone oil (cf. Fig. 2i). Conversely, lactitol/trehalose (cf. Fig. 2d) and saccharose (cf. Fig. 2h) irregularly fill the late wood tracheids, with some accumulation on the cell walls. The freeze-dried and PEG treated samples exhibit a more haphazard distribution within the tracheids. This pattern is notably apparent in the PEG1 sample (cf. Fig. 2e), where the consolidant selectively fills a region within a trachea. Whether earlywood or latewood is present, the cell lumens become filled, leading to uniform stabilization throughout the section by way of numerous points. The PEG2 treated sample demonstrates more substantial filling of the cell wall, although the cell lumens are less occupied (cf. Figs. 2f, 3).

### Raman analysis

In Raman imaging (cf. Fig. 3), the embedding agent appears in yellow. The distinctive lignin fluorescence often obscures the cellulose/hemicellulose regions. However, the red areas reveal a combination spectrum of the intrinsic and distinctive lignin and cellulose. The reference sample highlights that the embedding agent has effectively penetrated the cells (cf. Fig. 3i). It should be noted that the different nature of the samples posed various preparation challenges, resulting in surfaces of differing levels of flatness that required precise laser focusing.

However, the impact of the consolidants, as observed using synchrotron µCT, was supported by Raman microscopy, where all consolidants appeared in blue. It is worth noting that the consolidants used in the alcohol-ether-resin method could not be identified using synchrotron µCT or Raman imaging (cf. Figs. 2b, 3a). The sample was highly fragile, and there might be barely any consolidant within the investigated region. The significant deviation in the WPG measurements supports this observation. In the Kauramin 800® treated sample, some affinity of the melamine formaldehyde to the residual cell wall could be verified (cf. Fig. 3b). The deposition of consolidants in the cell lumen was observable in all other samples. Furthermore, the Raman analysis indicated that the sample conserved with lactitol/trehalose sustained reliable stabilization of both the lumen and cell wall (cf. Fig. 3c). PEG was present throughout the entire cell lumen and was noted in the residual cell wall structure in the PEG1 treated sample (cf. Fig. 3d). On the other hand, the PEG2-treated sample confirmed the coverage of the residual cell wall and filling of the cell lumen with porous freeze-dried PEG (cf. Fig. 3e). However, only a small amount of PEG was detected in the residual cell wall of the sample treated with PEG3 (cf. Fig. 3f). Saccharose was detected in the cell lumen (cf. Fig. 3g), while silicone oil was observed in both the residual cell wall and the cell lumen (cf. Fig. 3h).

## Discussion

The conservation methods exhibited differing degrees of effectiveness, suggesting that the mechanism of wood stabilization is contingent upon the method, including factors such as the concentration of the impregnation solution, molecular weight, and chemical composition. Additionally, the choice of drying method also played a significant role in the observed variations:

### Quantity of the conservation agents

The first question to be answered was, if the quantity of conservation agents integrated into the structure is a paramount factor for achieving volume stabilization. When examining the results for WPG and ASE presented in Fig. 1, it is evident that a connection exists between the quantity of stored consolidant, the WPG and the volume stabilization indicated by ASE, with the exception of the samples treated with silicone oil. While this samples displayed an increase in WPG of 58.8 ± 9. 5%, they only achieved an ASE of 73.2 ± 11.7%, deviating from the expected trend. Based on the data, there appears to be a saturation point reached beyond a WPG of 60%, whereby further volume stabilization was not effectively possible. Grattan^15^ recommends to adapt the PEG content proportionally with the fibre saturation point. Moderately preserved wood can be effectively stabilized with a smaller amount of conservation agent. In contrast, degraded wood requires a larger amount of conservation agent for stabilization, as the microporosity of the cell wall is increased due to degradation. This leads to a higher fiber saturation point in these materials. Further evidence supporting the correlation between stabilization and the amount of conservation agent can be found in related research^49^. In addition to the quantity of conservation agent used, the chemical affinity to the wood structure plays a role. A study has shown that organosilicons exhibited a link between the molecular weight of the particles and their absorption capabilities in wood. Smaller particles, with functional groups which enable an interaction with the wood polymers, demonstrated a higher tendency to penetrate and thus to stabilise the cell wall^40^.

### Mode of action of the conservation agent

The second question that guided our research was whether the mode of action (bulking/impregnation) significantly affects volume stability? This question is related to the third inquiry of whether the conservation agent is compatible with the cell wall. To answer this question, the consolidant must first be identified in the structure. To achieve the desired volume and dimensional stability, it is necessary to incorporate the resin into the microstructure of the cell wall^50^. Simply filling the cell volume is not enough.

Samples treated with alcohol-ether-resin showed shrinkage in the secondary cell walls (cf. Fig. 2b)^51^. In contrast, melamine formaldehyde forms a protective coating on the cell walls, providing substantial volume stability in the Kauramin 800® treated samples. It generates a three-dimensional framework by cross-linking throughout the structure. Moreover, melamine formaldehyde adheres to the wood through hydrogen bonding and the interactions between the lignin ring and melamine^52^. This elucidates the strong binding observed in the Raman examination between melamine formaldehyde and the cell wall. Formaldehyde is also reported to promote cross-linking in wood^38^.

The use of lactitol/trehalose treatment is recognised for providing noteworthy dimensional stability, especially in softwood species^30^, in line with the results of this investigation. Hoffmann's previous study^26^ produced less reliable outcomes, possibly owing to the inconsistent distribution of sugar alcohols.

The penetration of low molecular weight PEG into cellular walls is supported by numerous studies^53–55^, whereas high molecular weight PEG, including PEG 4000, demonstrates this capacity to a lesser extent^50^. These findings corroborate the current trend, but it is worth noting that PEG1 samples conserved with PEG 2000 exhibit superior volume stability. Discernible gaps can be seen between the middle lamellae and secondary cell walls of the late wood tracheids, indicating shrinkage in samples treated with PEG3 (PEG 400, 1500, and 4000) (cf. Fig. 3g). The comparison of PEG2 (10%) and PEG3 (approx. 15%) methods reveals that the concentration of low-molecular PEG 400 was not the decisive factor here. Instead, the total bath concentration played a more significant role, with PEG2 having a concentration of 35% and PEG3 having a concentration of 28%. Synchrotron µCT examinations of this particular sample revealed bright areas, indicating the presence of inorganic components from the soil environment^56,57^.

Besides bulked vessels it is reported that saccharose is penetrating the cell wall and bonds to e.g. to the remaining cellulose with considerable hydrogen bonding^49^. A considerable variability of the conserved vessels were documented here. The influence of the degradation state and nature of the remaining cell wall has also been observed in other studies^53,57^.

Nonetheless, successful silicone oil penetration did not mitigate shrinkage of the secondary cell wall^36^. This is apparent due to the presence of gaps in the cell wall. Despite previous reports of severe shrinkage and collapse, certain modifications to the treatment show promise for improvement^58^. Methyltrimethoxysilane (MTMS), as a small molecule, can permeate the cell wall, while longer-chain silanes encounter hindrances in doing so. These silanes are capable of crosslinking with each other through condensation and with alkoxy bonds present within the wood polymers^40^. In a study by Broda^59^, the mode of action of various organosilicons in the wood structure was examined and aligned with a model proposed by Norimoto^60^. This model indicates that organosilicons have the capability to fill both the cell wall and lumina.

### Drying method used for conservation

The effects of different drying methods on wood structure are apparent, with air-drying (cf. Fig. 2a) causing considerable harm. Conversely, solvent drying, as employed in the alcohol-ether resin process, aids in reducing drying-induced stress (cf. Fig. 2b)^61^. In situations where the wood is impregnated and the consolidant has settled, it can establish a robust internal framework within the delicate structure of middle lamellae and cell wall debris. This solid conservation agent can efficiently fill and stabilise degraded wood, protecting it from collapsing while air-drying (cf. Fig. 2c,d,i)^62^. Nonetheless, it should be noted that the accumulation of consolidants indicates uneven distribution and concentration in specific regions. Furthermore, the migration of the conservation agent during air-drying is observable, especially in samples treated with lactitol/trehalose and saccharose (cf. Fig. 2d,i).

As the solution evaporates, capillary forces lead to the transportation of the conservation agent. This results in the accumulation of conservation agents within the latewood tracheids with smaller volumes^57^. This accumulation is also apparent in the notably high bulk density of the samples treated with lactitol/trehalose and saccharose (cf. Fig. 1c).

The effects of freeze-drying are especially evident in the PEG1 and PEG2 samples (cf. Fig. 2b,c)^16,63,64^. In the initial stage of freeze-drying, the solution freezes, impacting mainly the water due to its hypoeutectic concentration, which occurs at approximately 0 °C. In this process, the consolidant is randomly distributed within the wood structure at the eutectic concentration when it freezes at the eutectic temperature. At this stage, the mixture solidifies and is no longer mobile. During the primary drying phase, water is eliminated through sublimation by decreasing the vapor pressure. In a freeze-drying facility, water vapour is drawn out of equilibrium and accumulates at the coldest point—the cold trap. This causes continuous sublimation of water from the structure until it is completely dried. The end product is a porous, solid high molecular weight PEG that retains its integrity even at room temperature. It is worth noting that imprints of ice crystals, which freeze earlier than the eutectic mixture, are visible within the freeze-dried PEG structure (cf. Fig. 2e–g). On other hand, PEG with low molecular weight remains in a liquid state and stays mobile at temperatures prevailed during the freeze-drying process^16,65^ (cf. Table 1).

## Conclusion

Conservation methods play an important role in the preservation of cultural heritage made of wood found in waterlogged environments. This study analyses the factors that contribute to differences in the efficacy of conservation agents that are observed in previous studies^20^. The first question was whether the amount of conservation agent introduced into the structure is an important factor in achieving volume stabilisation. The presence of the conservation agent in the wood could be indicated of all analysed conservation methods by both WPG and structural analysis. The volume stability measurements depend on the amount of conservation agent introduced into the wood with the exception of the silicone oil treated samples. Furthermore, this research has also provided insight into the reliability of these methods. Significant variability in the uptake of alcohol-ether resins and silicone oil was observed within this test series. This finding suggests that conserved artefacts in archaeological collections may not achieve uniform stabilization throughout their complete structure.

The second question was, whether the mode of action (bulking/impregnation) significantly influence volume stability. Tho answer this, the distribution of the conservation agent was evaluated. The three-dimensional synchrotron µCT analyses highlighted the conserved wood structure over a significant area, showing a rather uneven distribution of the conservation agents lactitol/trehalose, PEG and saccharose. Kauramin 800® result in bulking, while the sample treated with silicone oil showed full impregnation. The treatment with saccharose and lactitol/trehalose resulted in accumulation of these substances within the late wood. In contrast, the PEG and freeze-dried samples showed that only some tracheids were completely filled, while individual tracheids were generally only partially filled. Not all methods can be clearly assigned to one of the two modes of action described, except for Kauramin 800® and silicone oil. Instead, several mixed forms can be recognised here, namely impregnation and bulking, as well as a high degree of local variability in their characteristics. The investigation shows that the drying technique has an impact on the distribution of conservation agents in non-chemical reaction cure-based conservation methods. Air drying solutions result in the accumulation of conservation agents within latewood cells, whereas freeze-drying techniques produce a more even distribution. Therefore, it was not possible to establish a clear relationship between the mode of action of the conservation agents and their ability to stabilize the volume of the wood.

Finally, the study investigated the affinity of the conservation agents towards the degraded wood structure and its components. The Raman analyses revealed, that the conservation agents namely Kauramin 800®, lactitol/trehalose, PEG and silicone oil were deposited in the cell wall. However, further investigations are required to determine the chemical compatibility of the conservation agents with the cell wall.

These findings enhance our understanding of the structure of conserved wood. Stabilising waterlogged wood is a complex process that involves intricate chemical interactions between the wood and conservation agents. The degree of degradation and the morphological features of wood anatomy significantly impact the process. This research suggests the inclusion of other wood genus. However, this study’s findings are crucial in improving our comprehension of properties, particularly in relation to the mechanical stability of conserved museum artefacts. The weight of these objects affects their handling, transport, exhibition, and stabilization due to their composite nature, comprising archaeological wood mixed with consolidants.

## Data Availability

3D meshes of wood samples are available here: 10.5281/zenodo.7950582.

## References

[1] Hocker, E. *Preserving Vasa* (Archetype Publications, 2018).

[2] Björdal, C. G. Microbial degradation of waterlogged archaeological wood. *J. Cult. Herit.* **13**, 118–122 (2012). 10.1016/j.culher.2012.02.003

[3] Singh, A. P., Kim, Y. S. & Chavan, R. R. Advances in understanding microbial deterioration of buried and waterlogged archaeological woods: a review. *Forests* **13**, 394 (2022). 10.3390/f13030394

[4] Pournou, A. *Biodeterioration of wooden cultural heritage: organisms and decay mechanisms in aquatic and terrestrial ecosystems* (Springer, 2020).

[5] Blanchette, R. A. A review of microbial deterioration found in archaeological wood from different environments. *Int. Biodeterior.*10.1016/S0964-8305(00)00077-9 (2000). 10.1016/S0964-8305(00)00077-9

[6] Pedersen, N. B., Björdal, C. G., Jensen, P. & Felby, C. Bacterial degradation of archaeological wood in anoxic waterlogged environments. In *Stability of Complex Carbohydrate Structures. Biofuel, Foods, Vaccines and Shipwrecks* Vol. 341 (ed. Harding, S. E.) 160–187 (Royal Society of Chemistry, 2013).

[7] Pedersen, N. B., Łucejko, J. J., Modugno, F. & Björdal, C. Correlation between bacterial decay and chemical changes in waterlogged archaeological wood analysed by light microscopy and Py-GC/MS. *Holzforschung* **75**, 635–645 (2021). 10.1515/hf-2020-0153

[8] Hawley, L. F. Wood–liquid relations. *Tech. Bull.* **248**, 1–34 (1931).

[9] Jiachang, C., Donglang, C., Jingen, Z., Xia, H. & Shenglong, C. Shape recovery of collapsed archaeological wood ware with active alkali-urea treatment. *J. Archaeol. Sci.* **36**, 434–440 (2009). 10.1016/j.jas.2008.09.027

[10] Barbour, R. J. & Leney, L. Shrinkage and collapse in waterlogged archaeological wood: Contribution III Hoko River series. In *Proceedings of the ICOM Waterlogged Wood Working Group Conference. Ottawa* (eds. Grattan, D. & McCawley, J. C.) 208–225 (1981).

[11] Herbst, C. F. Om bevaring af Oldsager af Traetfundet Torfmoser. *Antiqu. Tidskr.* **1858–60**, 174–176 (1881).

[12] Broda, M. & Hill, C. A. S. Conservation of waterlogged wood—Past, present and future perspectives. *Forests* **12**(9), 1193. 10.3390/f12091193 (2021). 10.3390/f12091193

[13] EN 15898: 2011. DIN-Taschenbuch 409 Erhaltung des kuturellen Erbes (Beuth, 2014)

[14] Grattan, D. W., Clarke, R. W. Conservation of waterlogged wood. In Conservation of Marine Archaeological Objects (ed. C. Pearson) 164–206 (Butterworths, 1987).

[15] Grattan, D. W. A practical comparative study of several treatments for waterlogged wood. *Stud. Conserv.* **27**, 124–136 (1982). 10.1179/sic.1982.27.3.124

[16] Schnell, U. & Jensen, P. Determination of maximum freeze–drying temperature for PEG-Impregnated archaeological wood. *Stud. Conserv.* **52**, 50–58 (2007). 10.1179/sic.2007.52.1.50

[17] Walsh-Korb, Z. & Avérous, L. Recent developments in the conservation of materials properties of historical wood. *Progress Mater. Sci.* **102**, 167–221 (2019). 10.1016/j.pmatsci.2018.12.001

[18] *Massenfunde in archäologischen Sammlungen*. www.rgzm.de/kur (2022).

[19] Stelzner, I. *et al.* Evaluation of conservation methods for archaeological wet wood with structured light 3D scanning and µ-CT. In *Proceedings of the 15th ICOM-CC Group on Wet Organic Archaeological Materials Conference, Mainz 2023.* (eds. Hovmand, I. et al.) 96–105 (ICOM-CC, 2023).

[20] Stelzner, J. *et al.* Stabilisation of waterlogged archaeological wood: the application of structured-light 3D scanning and micro computed tomography for analysing dimensional changes. *Herit. Sci.* **10**, 60 (2022). 35578712 10.1186/s40494-022-00686-6 PMC9098614

[21] Macchioni, N., Pizzo, B., Capretti, C. & Giachi, G. How an integrated diagnostic approach can help in a correct evaluation of the state of preservation of waterlogged archaeological wooden artefacts. *J. Archaeol. Sci.* **39**, 3255–3263 (2012). 10.1016/j.jas.2012.05.008

[22] High, K. E. & Penkman, K. E. H. A review of analytical methods for assessing preservation in waterlogged archaeological wood and their application in practice. *Herit. Sci.* **8**, 83 (2020). 10.1186/s40494-020-00422-y

[23] Jensen, P. & Gregory, D. J. Selected physical parameters to characterize the state of preservation of waterlogged archaeological wood: A practical guide for their determination. *J. Archaeol. Sci.* **33**, 551–559 (2006). 10.1016/j.jas.2005.09.007

[24] de Jong, J. The conservation of waterlogged timber at Ketelhaven (Holland). In *4th ICOM-CC Triennial Meeting Venice Italy 13–18 October 1975* (ed. ICOM Committee for Conservation) 75/8/1-1–9 (ICOM-CC, 1975).

[25] Cook, C. & Grattan, D. A method of calculation the concentration of PEG for freeze–drying waterlogged wood. In *Proceedings of the 4th ICOM-CC Group on Wet Organic Archaeological Materials Conference* (ed. Hoffmann, P.) 239–252 (1990).

[26] Hoffmann, P. *Conservation of Archaeological Ships and Boats: Personal Experiences* (Archetype Publications, 2013).

[27] Schmidt-Ott, K., André, C. & Bader, M. Fishing for stability: conserving a fish trap in a block excavation by the alcohol-ether-resin method. In *Wet Organic Archaeological Materials 2019. Proceedings of the 14th ICOM-CC Wet Organic Archaeological Materials Working Group Interim Meeting, Portsmouth 2019,* 322–327 (ICOM-CC, 2022).

[28] Schmidt-Ott, K., André, C., Liengme, G. & Hildbrand, E. Optimisation of the alcohol-ether-resin method for wood and composite objects. In *Proceedings of the 15th ICOM-CC Group on Wet Organic Archaeological Materials Conference, Mainz 2023.* (eds. Hovmand, I. et al.) 145–152 (ICOM-CC, 2023).

[29] Wittköpper, M. Der aktuelle Stand der Konservierung archäologischer Naßhölzer mit Melamin/Aminohärzen am Römisch-Germanischen Zentralmuseum. *Arbeitsblätter Restaur.* **31**, 227–283 (1998).

[30] Imazu, S. & Morgos, A. Conserving waterlogged wood using sugar alcohols and comparison the effectiveness of Lactitol MC, Saccharose and PEG 4000 treatment. In *Proceedings of the 6th ICOM Group on Wet Organic Archaeological Materials Conference, York, 1996* (eds. Hoffmann, P., Daley, T., Grant, T. & Spriggs, J. A.) 235–255 (ICOM-CC, 1997).

[31] Imazu, S. & Morgos, A. Lactitol conservation of a 6 m long waterlogged timber coffin. In *Proceedings of the 7th ICOM-CC Group on Wet Organic Archaeological Materials Conference, Grenoble, 1998* (eds. Bonnot-Diconne, C., Hiron, X., Tran, K. & Hoffmann, P.) 210–214 (ICOM-CC, 1999).

[32] Imazu, S. & Morgós, A. An improvement on the Lactitol MC conservation method used for the conservation of archaeological waterlogged wood (The conservation method using lactitol MC and trehalose mixture). In *Proceedings of the 8th ICOM Group on Wet Organic Archaeological Materials Conference, Stockholm 2001* (eds. Hoffmann, P., Spriggs, J. A., Grant, T., Cook, C. & Recht, A.) 413–428 (ICOM-CC, 2002).

[33] Jensen, P., Petersen, A. H. & Straetkvern, K. From the Skuldelev to the Roskilde ships—50 years of shipwreck conservation at the National Museum of Denmark. In *Shipwrecks 2011. Chemistry and Preservation of Waterlogged Wooden Shipwrecks* (ed. Ek, M.) 14–20 (ICOM-CC, 2011).

[34] Hoffmann, P. Zur Naßholzkonservierung mit Zucker am Deutschen Schiffahrtsmuseum eine Bilanz. *Arbeitsblätter Restaur.* **10**, 231–241 (1996).

[35] Dumkow, M. & Preuß, H. Konservierung von Naßholz mit Rübenzucker. *Arbeitsblätter Restaur.* **23**, Gruppe-8 (1990).

[36] Hamilton, D. L. *Methods of conserving archaeological material from underwater sites*. vol. Conservation of archaeological resources I (2010).

[37] Toloczko, S. & Crawshaw, A. Not all silicone oils are born equal! In *Proceedings of the 15th ICOM-CC group on wet organic archaeological materials conference, Mainz 2023.* (eds. Hovmand, I. et al.) 250 (ICOM-CC, 2023).

[38] Awais, M. *et al.* Wood–water relations affected by anhydride and formaldehyde modification of wood. *ACS Omega* **7**, 42199–42207 (2022). 36440166 10.1021/acsomega.2c04974 PMC9685604

[39] Cutajar, M., Braovac, S., Stockman, R. A., Howdle, S. M. & Harding, S. E. Evaluation of two terpene-derived polymers as consolidants for archaeological wood. *Sci. Rep.* **13**, 3664 (2023). 36871097 10.1038/s41598-023-29785-5 PMC9985608

[40] Broda, M. *et al.* Organosilicons of different molecular size and chemical structure as consolidants for waterlogged archaeological wood—A new reversible and retreatable method. *Sci. Rep.* **10**, 2188 (2020). 32042023 10.1038/s41598-020-59240-8 PMC7010770

[41] Wood Handbook, Wood as an Engineering Material (Forest Products Laboratory, 2010).

[42] Grattan, D. W. & McCawley, J. C. The potential of the Canadian winter climate for the freeze–drying of degraded waterlogged wood. *Stud. Conserv.* **23**, 157–167 (1978). 10.1179/sic.1978.021

[43] Wittköpper, M. *et al.* The KUR (conservation and restauration) project—A comparison of different methods to preserve waterlogged wood. In *Proceedings of the 12th ICOM-CC Group on Wet Organic Archaeological Materials Conference, Istanbul 2013* (eds. Grant, T. & Cook, C.) 134–143 (ICOM-CC, 2016).

[44] Cecilia, A. *et al.* LPE grown LSO: Tb scintillator films for high-resolution X-ray imaging applications at synchrotron light sources. *Nucl. Instrum. Methods Phys. Res. Sect. Accel. Spectrometers Detect Assoc. Equip.* **648**, 321–323 (2011). 10.1016/j.nima.2010.10.150

[45] Vogelgesang, M. *et al.* Real-time image-content-based beamline control for smart 4D X-ray imaging. *J. Synchrotron Radiat.* **23**, 1254–1263 (2016). 27577784 10.1107/S1600577516010195

[46] Vogelgesang, M., Chilingaryan, S., Rolo, T. dos Santos & Kopmann, A. UFO: A scalable GPU-based image processing framework for on-line monitoring. In *2012 IEEE 14th International Conference on High Performance Computing and Communication & 2012 IEEE 9th International Conference on Embedded Software and Systems* 824–829 (2012). 10.1109/HPCC.2012.116.

[47] Faragó, T. *et al.* Tofu: A fast, versatile and user-friendly image processing toolkit for computed tomography. *J. Synchrotron Radiat.* **29**, 916–927 (2022). 35511025 10.1107/S160057752200282X PMC9070706

[48] Paganin, D., Mayo, S. C., Gureyev, T. E., Miller, P. R. & Wilkins, S. W. Simultaneous phase and amplitude extraction from a single defocused image of a homogeneous object. *J. Microsc.* **206**, 33–40 (2002). 12000561 10.1046/j.1365-2818.2002.01010.x

[49] Parrent, J. M. The conservation of waterlogged wood using sucrose. *Stud. Conserv.* **30**, 63–72 (1985). 10.1179/sic.1985.30.2.63

[50] Altgen, M. *et al.* Distribution and curing reactions of melamine formaldehyde resin in cells of impregnation-modified wood. *Sci. Rep.* **10**, 3366 (2020). 32098986 10.1038/s41598-020-60418-3 PMC7042241

[51] Bräker, O. U. *et al.* Zum derzeitigen Stand der Nassholzkonservierung. Diskussion der Grundlagen und Resultate eines von Fachlaboratorien 1976–1978 durchgeführten Methodenvergleiches. *Z. Für Schweiz. Archaeol. Kunstgesch.* **36**, 97–145 (1979).

[52] Spinella, A. *et al.* Solid state NMR investigation of the roman acqualadroni rostrum: tenth year assessment of the consolidation treatment of the wooden part. *Cellulose* **28**, 1025–1038 (2021). 10.1007/s10570-020-03563-2

[53] Hoffmann, P. On the stabilization of waterlogged oakwood with PEG: Molecular size versus degree of degradation. In *Proceedings of the 2nd ICOM Waterlogged Wood Working Group conference, Grenoble, 28–31 August 1984* (eds. Ramiere, R. & Colardelle, M.) 95–115 (Centre d´étude et de traitement de bois d’eau and ICOM-CC WOAM, 1985).

[54] Bilz, M., Grant, T. & Young, G. Treating waterlogged basketry: a study of polyethylene glycol penetration into the inner bark of western red cedar. In *Proceedings of the 7th ICOM Group on Wet Organic Archaeological Materials Conference* (eds. Bonnot-Diconne, C., Hiron, X., Khoi Tran, Q. & Hoffmann, P.) 249–253 (ICOM-CC, 1999).

[55] Han, L., Guo, J., Tian, X., Jiang, X. & Yin, Y. Evaluation of PEG and sugars consolidated fragile waterlogged archaeological wood using nanoindentation and ATR-FTIR imaging. *Int. Biodeterior. Biodegrad.* **170**, 105390 (2022). 10.1016/j.ibiod.2022.105390

[56] Scott, D. A. & Eggert, G. The vicissitudes of vivianite as pigment and corrosion product. *Stud. Conserv.* **52**, 3–13 (2007). 10.1179/sic.2007.52.Supplement-1.3

[57] Stelzner, I., Stelzner, J., Gwerder, D., Martinez-Garcia, J. & Schuetz, P. Imaging and assessment of the microstructure of conserved archaeological pine. *Forests* **14**, 211 (2023). 10.3390/f14020211

[58] Kavvouras, P. K., Kostarelou, C., Zisi, A., Petrou, M. & Moraitou, G. Use of silanol-terminated polydimethylsiloxane in the conservation of waterlogged archaeological wood. *Stud. Conserv.* **54**, 65–76 (2009). 10.1179/sic.2009.54.2.65

[59] Broda, M., Jakes, J. E., Li, L. & Antipova, O. A. Archeological wood conservation with selected organosilicon compounds studied by XFM and nanoindentation. *Wood Sci. Technol.* **57**, 1277–1298. 10.1007/s00226-023-01503-4 (2023). 10.1007/s00226-023-01503-4 PMC11845243 39990724

[60] Norimoto, M. Chemical modification of wood. In *Wood and cellulose chemistry* (eds. Hon, DN-S & Shirashi, N.) 573–598 2nd edn. (Marcel Dekker, 2001).

[61] Christensen, B. B. Om konservering af mosefundne trægenstande - Conservation of waterlogged wood. *Aarbøger Nord. Oldkynd. Og Hist.* 22–62 (1951).

[62] Jensen, P., Straetkvern, K., Bojesen-Koefoed, I. & Gregory, D. Freeze–drying of archaeological waterlogged wood. in *Conservation of archaeological ships and boats - personal experiences* (ed. Hoffmann, P.) 105–118 (Archetype Publ, 2013).

[63] Stelzner, I. *Zur Nassholzkonservierung Bestimmung prozessrelevanter Eigenschaften für die Gefriertrocknung* (Staatliche Akademie der Bildenden Künste, 2017).

[64] Oetjen, G.-W. & Haseley, P. *Freeze–drying* (Wiley-VCH, 2004).

[65] Wiesner, I. & Gieseler, H. Freeze–dry microscopy—Real-time observation of the drying process. In *Proceedings of the 12th ICOM-CC Wet Organic Archaeological Materials Conference, Istanbul 2013* (eds. Cook, C. & Grant, T.) 417–424 (ICOM-CC, 2016).

